# Structure Evolution of Epoxidized Natural Rubber (ENR) in the Melt State by Time-Resolved Mechanical Spectroscopy

**DOI:** 10.3390/ma13040946

**Published:** 2020-02-20

**Authors:** Rossella Arrigo, Leno Mascia, Jane Clarke, Giulio Malucelli

**Affiliations:** 1 Department of Applied Science and Technology and local INSTM Unit, Politecnico di Torino, Viale Teresa Michel 5, 15121 Alessandria, Italy; giulio.malucelli@polito.it; 2 Department of Materials, Loughborough University, Loughborough LE11 3TU, UK; l.mascia@lboro.ac.uk (L.M.); J.Clarke@lboro.ac.uk (J.C.)

**Keywords:** time-resolved mechanical spectroscopy, rheological properties, transient-state polymers, linear viscoelastic behavior, epoxidized natural rubber

## Abstract

In this work, time-resolved mechanical spectroscopy (TRMS) was used to accurately characterize the rheological behavior of an epoxidized natural rubber (ENR) containing 25 mol% of epoxy groups. Conventional rheological tests are not suitable to characterize with accuracy the frequency-dependent linear viscoelastic behavior of materials, such as ENR, in a transient configurational state. For this reason, TRMS was used to determine the true rheological behavior of ENR, as well as to gain some insights into the changes of its macromolecular architecture under the dynamic conditions experienced during the measurements. The constructed master curves for the moduli revealed a gradual transition of the ENR rheological state from liquid-like to solid-like through the formation of an “elastic gel” throughout the bulk of the polymer. Furthermore, the evolution of the stress relaxation modulus revealed a slow relaxation mechanism, resulting from thermally activated reactions in the molten state attributed to the formation of crosslinks. Finally, the crosslink density evolution was estimated from the TRMS data and compared with results derived from equilibrium solvent-swelling measurements. These demonstrated the accuracy of the TRMS data in the prediction of the structural changes that can take place in polymers during processing.

## 1. Introduction

Epoxidized natural rubber (ENR) is a non-fossil fuel-based elastomer derived from the chemical modification of natural rubber (epoxidation) in order to increase the polarity of groups along the polymer chains, while maintaining most of the characteristics of its native counterpart [1,2,3]. Notable among these is the ability of ENR to crystalize under strain up to 50 mol% of epoxidation level and to exhibit “good” mechanical properties [4]. Compared to natural rubber, ENR shows several other advantages, such as low gas permeability, good adhesion, good damping and wet grip performance, and oil resistance [5,6,7]. Additionally, the presence of epoxy groups randomly distributed along the polymer chains enhances the compatibility of this modified natural rubber with polymers bearing polar groups and facilitates the dispersion of fillers without the need of coupling agents [8]. ENR has also been used as a compatibilizer or property modifier in polymer blends [9] and composites [10] due to the ability of the epoxy groups in ENR to interact with functional groups present on the filler surface, thereby avoiding the formation of particle agglomerates within the host matrix [11]. This can be exemplified by the work of Wang et al. [12], exploiting the interfacial reactions, involving a ring-opening mechanism, between the epoxy groups of ENR and the silanol groups present on a silica surface. The highly enhanced rubber–filler interactions have given rise to large improvements in filler dispersion and in the mechanical properties of the resulting composites. Recently, various attempts have been made to prepare composites fully based on ENR containing a variety of fillers, including silica [13], lignin [14], and carbon nanotubes [15,16]. Experiments have also been reported on the use of ENR as a rubber matrix in composites containing an innovative geopolymer derived from fly ash waste from electric power-generating plants [17]. The composite exhibited a good dispersion of the filler in the ENR matrix, attributed to chemical interactions between functional groups in the ENR and polar functional groups on the filler surface.

The macromolecular architecture of polymers and polymer-based multiphase systems can be characterized from an examination of their rheological behavior [18] due to the singularity of the relationship between macromolecular motions and the relaxation dynamics of polymer chains [19,20]. This is particularly evident in the rheological response at low frequency, which reveals distinct information about the macromolecule dynamics due to the possibility of probing the slow relaxations of the predominant portion of the material [21]. However, since measurements by conventional rheological tests, carried out at fixed frequency, require very long experimentation time, the analysis is only suitable for polymers exhibiting a high structural stability within the duration of the tests. It has also been shown that conventional frequency sweep tests are not accurate when an event involving a mutation (i.e., a structural modification affecting the mobility of the macromolecules) takes place during the measurement within a time interval that exceeds the natural relaxation time of the polymer [22]. Mutation of the macromolecular architecture can be related to various events, such as changes in molecular weight and molecular weight distribution [22] or in the physical or chemical connectivity between polymer molecules within a gel [23]. Thermally induced phase separation can take place in polymer blends or block copolymers [24], while flocculation of nanoparticles can be experienced in polymer-based composites [25], and gelation phenomena [26] or fast degradation reactions can occur when measurements are performed at high temperatures (melt state) [27].

Several studies over the last two decades have shown that the “true” rheological behavior of the so-called transient polymers can be obtained using time-resolved mechanical spectroscopy (TRMS) [28,29]. In time-resolved rheological measurements, frequency-dependent rheological functions are obtained by subjecting the material to successive frequency sweeps over a realistic time schedule for this type of evaluation. The data collected for each frequency are then plotted as a function of the acquisition time and extrapolated to “zero” time to obtain a fundamental rheological parameter for the material [29], alongside the time dependency function for structure evolution. This procedure has often been used to characterize the thermal and thermo-oxidative degradation of polymers, nanocomposites, and blends [24,27,28].

To the best of the authors’ knowledge, this is the first example of the use of TRMS to evaluate the “molecular and microstructure” evolution of a rubber-like material in the melt state under dynamic loading conditions. Several reports dealing with the evaluation of the melt behavior of ENR and ENR-based blends and composites are available in the literature [30,31,32,33]. These deal primarily with the analysis of the rheological functions as a means of optimizing the processing conditions [6] and for the evaluation of the efficiency of curing agents [34]. However, the intrinsic latent structural instability of ENR, arising from the insertion of epoxy groups along the polymer chains, has not hitherto been studied by rheological measurements. Accordingly, the aim of this study was to evaluate the structural stability of a typical epoxidized natural rubber by TRMS. To this end, the evolution of the related rheological functions was examined by monitoring the development of a macromolecular network from the polymer relaxation spectrum.

## 2. Materials and Methods

### 2.1. Materials

In this work, an epoxidized natural rubber (ENR25) containing 25 mol% of epoxy groups was employed. (Epoxyprene 25, manufactured by Muang Mai Guthrie Public Limited Company of Muang, Thailand and donated by Tun Abdul Razak Research Centre). Main characteristics: Mooney viscosity in the range of 70–100 MU, glass transition temperature of −45 °C. 

### 2.2. Processing

Processing of the ENR25 samples was carried out with the aid of a Brabender-Plastograph mixer operating at 90 °C and 100 rpm for 10 min. Specimens for the rheological characterization were obtained by compression molding, using a laboratory press (Collin Teach Line 200T), working at 90 °C with an applied pressure of 100 bar for 2 min.

### 2.3. Characterization

Rheological measurements were performed using a strain-controlled rheometer (ARES, TA Instrument, New Castle, DE, USA) with a parallel plate geometry and a plate diameter of 25 mm. Preliminary time sweep tests for the rheological characterization of the material were carried out at 180 °C over a wide range of frequencies. TRMS tests were performed in air at 180 °C. The samples were subjected to 15 repeated frequency sweeps from 10^−1^ to 10^2^ rad/s. Storage (*G*’) and loss (*G*”) moduli and loss angle tangent (*tanδ*) were recorded during the measurements. In all measurements, the strain amplitude was fixed at 10%, which is within the linear viscoelastic region (established from preliminary strain sweep tests carried out at 180 °C and ω = 1 rad s^−1^). The gap between the plates was set to 1 mm.

The crosslink density of the samples at several stages of the evaluation was determined from equilibrium solvent-swelling measurements (in toluene at room temperature), applying the modified Flory–Rehner equation for tetra functional networks, previously used by other workers [35,36]. In brief, the samples (thickness = 2 mm) were initially weighed (*m_i_*) and immersed in toluene for 72 h at room temperature. Then, the swollen samples were dried to remove the solvent excess and weighted (*m_g_*). The remaining solvent was eliminated by drying in air for 6 days and then in an oven at 80 °C for 3 h. Finally, the samples were weighted (*m_s_*), and volume fractions of ENR (V_ENR_) in the samples at equilibrium swelling were determined as follows:

$$V_{ENR}=\;\frac{1}{1+swelling\;ratio}$$

$$Swelling\;ratio=\frac{m_{g}-m_{s}}{m_{s}}\times \frac{\rho_{e}}{\rho_{s}}$$

where *ρ_e_* and *ρ_s_* are the densities of elastomer samples and solvent, respectively.

## 3. Results and Discussion

**Figure 1:**
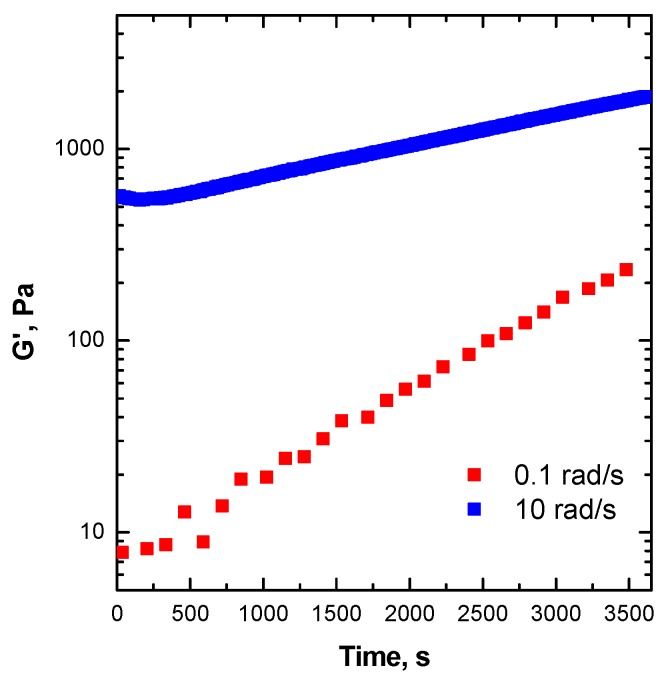
Time sweep measurements at two angular frequencies carried out at 180 °C for epoxidized natural rubber (ENR) 25. Preliminary tests.

In order to verify the microstructural stability of ENR in the molten state, the time evolution of the viscoelastic parameters was recorded at a constant temperature (180 °C). The results collected from experiments at different frequencies are plotted in Figure 1. The rheological curves show that a significant increase of the storage modulus (G’) took place over the duration of the experiments, particularly for tests performed at low frequency, which is indicative of some modification in the macromolecular architecture of ENR occurring during the measurement [37]. It is worth noting that the variation of G’ as a function of time was more pronounced in the low-frequency region, i.e., the slow macromolecular dynamics region, where large portions of polymeric material exhibited a large relaxation time.

**Figure 2:**
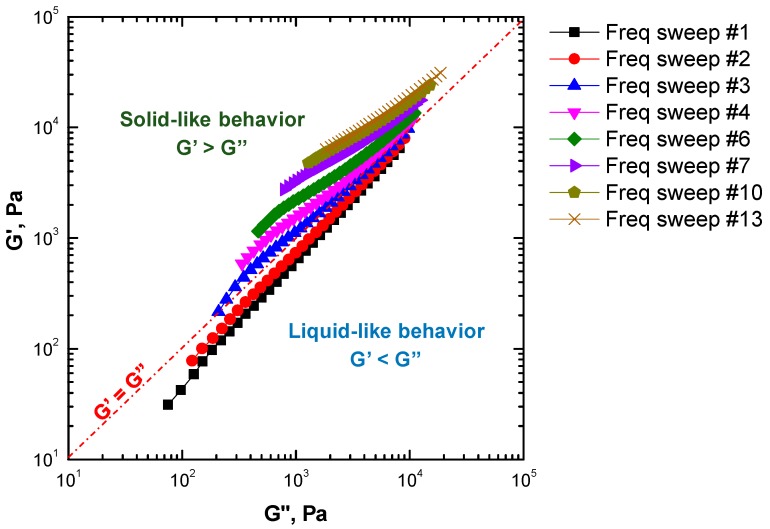
Curves of G’ vs. G” at 180 °C for ENR25 subjected to subsequent frequency sweeps.

The results obtained from the time sweep tests revealed the instability of ENR during rheological measurements at the selected temperature (Figure 1). These confirmed that conventional frequency sweep tests could not provide valuable and meaningful data for this polymer. Therefore, an accurate evaluation of the frequency-dependent linear viscoelastic behavior of ENR could be obtained using time-resolved mechanical spectroscopy. More specifically, the sample was subjected to several subsequent frequency sweep measurements. The obtained results are shown in Figure 2, in the form of a Han plot [38]. This type of plot is very useful for monitoring changes in the macromolecular architecture of polymers insofar as it can be used to categorize the events in two different physical-state regimes, i.e., a liquid-like state, characterized by G’ values lower than G”, and the consecutive solid-like state, where the G’ values are higher than G”. At the beginning of the test, ENR showed a liquid-like rheological behavior (G’ < G”), typical of unfilled polymers whose macromolecules are fully relaxed when in the molten state [39]. After about three frequency sweep tests, a progressive remarkable solid-like behavior appeared, recognizable by the increase of G’ with respect to G” and the gradual flattening of the curves. The progressive dominant elastic behavior as a function of time is indicative of the occurrence of some thermally activated phenomena causing the evolution of the initial ENR microstructure, which affected the polymer viscoelastic behavior.

**Figure 3:**
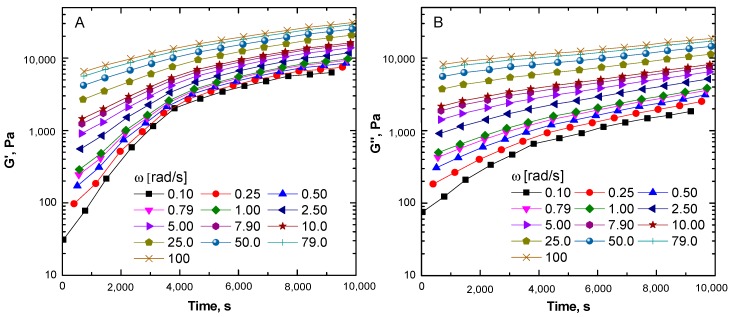
Time-resolved mechanical spectroscopy (TRMS) sweeps for ENR25 at 180 °C: storage modulus (**A**) and loss modulus (**B**).

To gain a better understanding of the structural changes, the data collected during the subsequent frequency sweeps were split according to individual frequencies and re-arranged as a function of time in order to produce moduli curves, shown in Figure 3. Both G’ and G” at each frequency represent the characteristic viscoelastic behavior as a function of time. The graphs clearly revealed more pronounced effect of the macromolecular structure evolution on G’ than on G”. This was expected, since the storage modulus is more sensitive to the formation of cross-links in the structure than the loss modulus [40]. It is important to note that the rise of the two moduli with time was more pronounced at low frequencies, which is the condition for probing the slow relaxations inherent to a large portion of a macromolecular network. Conversely, the molecular portion that relaxes faster is probed at high frequency, in concordance with the response of a small fraction of polymer network, which is the condition in which the growth of the two functions (G’ and G”) is much blander. Irrespectively of the tested frequency, the results provided the evidence for the occurrence of a thermally activated process leading to the restriction of the dynamics of the ENR macromolecules.

**Figure 4:**
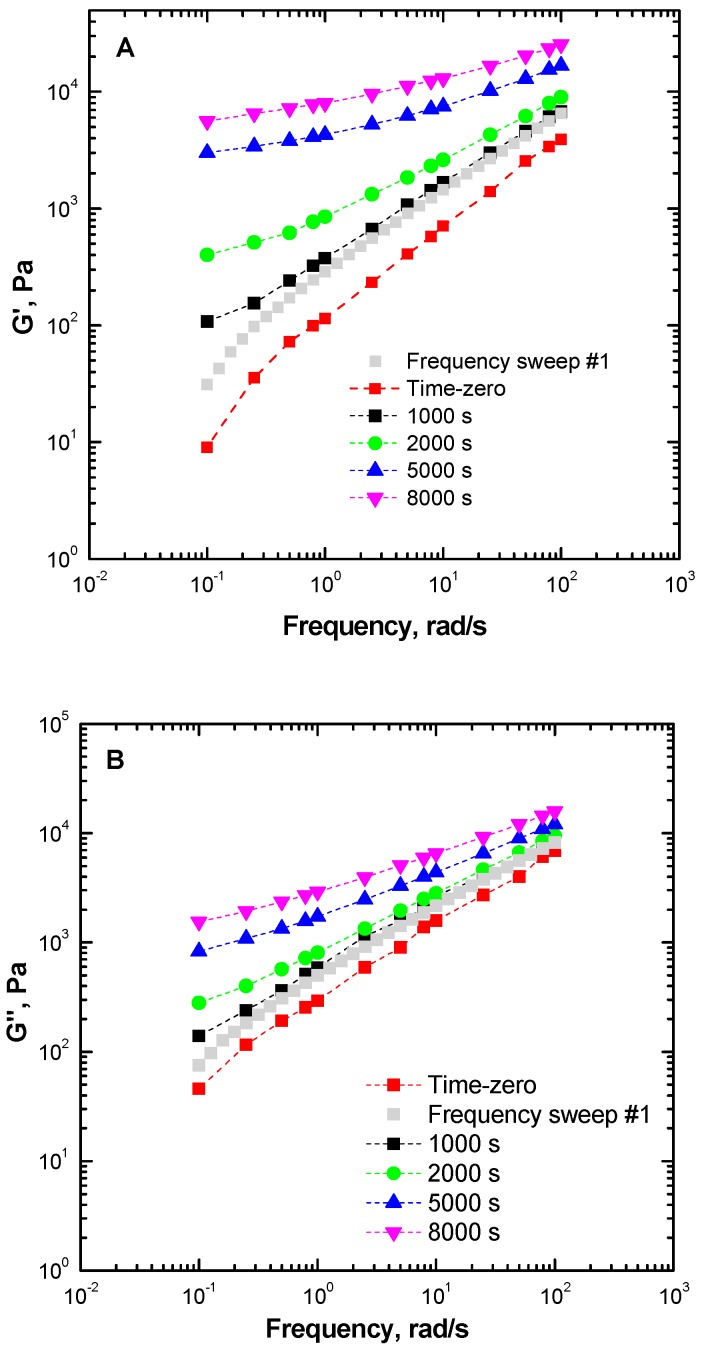
Isochronal storage modulus (**A**) and loss modulus (**B**) of ENR25 at 180 °C, collected at different times during the TRMS tests.

To isolate the effect of the microstructural alterations affecting the resulting changes in viscoelastic behavior, the “isochronal” moduli values for G’ and G” were calculated as the intercept of the vertical line at each time t and by interpolating the experimental data. From this interpolation procedure, it was possible to estimate the modulus values at time zero ($G_{0}^{\prime }$) as follows:

(1) $$\log G_{0}^{\prime }=\log G_{1}^{\prime }-\;m_{1,2}t_{1}$$

(2) $$m_{1,2}=\;\frac{\log G_{2}^{\prime }-\log G_{1}^{\prime }}{\Delta t_{1,2}}$$

where $G_{1}^{\prime }$ and $G_{2}^{\prime }$ are the modulus values at times *t*_1_ and *t*_2_, and *m*_1,2_ is the slope of the curve *G*(*t*) in the terminal region [28]. Figure 4 shows the master curves of the isochronal moduli at different times during the TRMS experiment, alongside with the extrapolated time-zero rheological parameters. The results of a single frequency sweep (which described the behavior probed in conventional rheological tests) obtained on a fresh sample (gray squares) are also shown to obtain a quantitative evaluation of the structural evolution in ENR.

Significant differences are observed when comparing the curve obtained after the first frequency sweep test and the extrapolated “time-zero” parameters. The two curves are expected to overlap if the material does not undergo any structural changes during the measurement, i.e., if the polymer is thermally stable. In examining the data in Figure 4, one notes a remarkable overestimation of the modulus values for the case of a single frequency scan performed on a fresh sample (conditions used in conventional rheological tests), as well as a significant change in the shape of the trendline for the frequency function. Moreover, the time evolution of the rheological parameters obtained by TRMS displays a more prominent “elastic nature” of the rheological behavior of the ENR elastomer with increasing time, alongside a gradual decrease of the gradient of the curves in the terminal (i.e., low-frequency) region as a function of time. More specifically, at time zero, both moduli curves exhibit the typical frequency dependence of polymers, for which *G’* and *G”* are proportional to ω^2^ and ω, respectively. Both moduli curves tend to become frequency-independent in the terminal region, which is indicative of a transition of the viscoelastic response from liquid-like to solid-like [41]. It should also be noted that the observed gradual growth of the rheological parameters of ENR is consistent with a progressive appearance of a long relaxation mechanism, which can be related with the formation of a three-dimensional macromolecular network (gelation phenomenon) [42,43,44].

**Figure 5:**
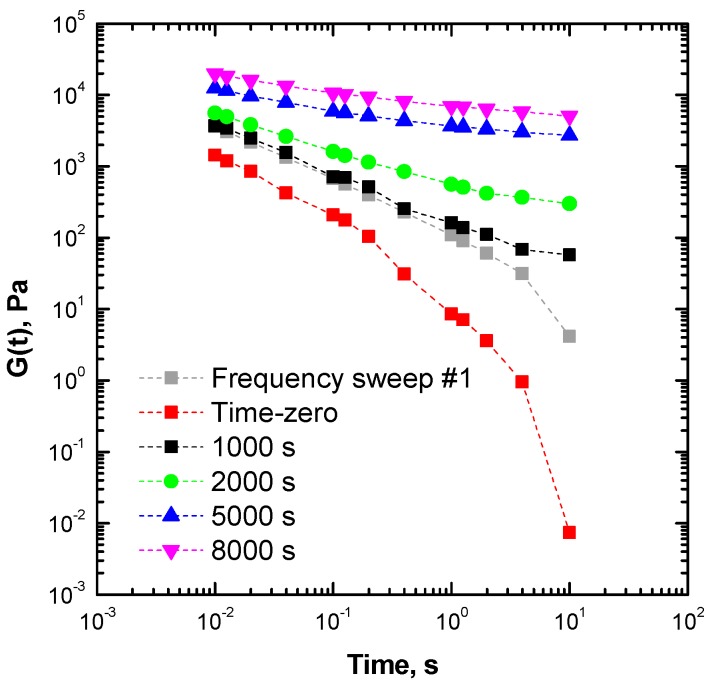
Stress relaxation curves for structural modifications of ENR25 at 180 °C.

To evaluate the relaxation dynamics of ENR25 macromolecules and to confirm the establishment of a different slow relaxation mechanism, stress relaxation curves (converting $G^{\prime }\left(\omega \right)$ and$\;G^{\prime \prime }\left(\omega \right)$ into G(t) values) were constructed at different time intervals from the TRMS rheological data, through the following formula suggested by Schwarzl [45,46]:

(3) $$\;G\left(t\right)=G^{\prime }\left(\omega \right)-aG^{\prime \prime }\left(\frac{\omega }{2}\right)+bG^{\prime \prime }\left(\omega \right)$$

where *a* and *b* are constants derived from the numerical interpolation of the experimental data [47]. The results, shown in Figure 5, confirmed a significant time dependence of the relaxation dynamics of ENR. More specifically, the data extrapolated at time zero predicted a liquid-like behavior fully capable of relaxation, though a gradual decrease of *G*(*t*) was observed, as indicated by the equilibrium value tending to zero at long times.

The resulting changes in the ENR25 relaxation spectrum can again be related to the evolution of a solid-like rheological behavior, associated with the initial formation (gel) and subsequent densification of the macromolecular network, which progressively restricted the dynamics of the ENR25 network, slowing down the motion and preventing a full relaxation of the macromolecules [48]. 

**Figure 6:**
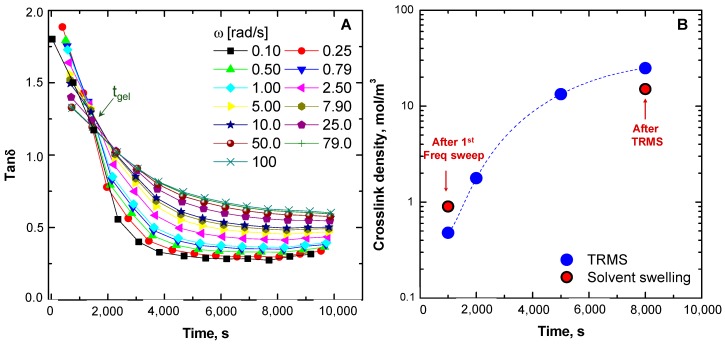
Evolution of *tanδ* during the TRMS tests (**A**) and crosslink density (from TRMS data and from solvent-swelling experiments) as a function of time (**B**).

In order to gain further insight into the significance of the rheological data of the TRMS tests and to confirm the formation of a gel during the tests, we also used the well-established Winter–Chambon method to characterize the changes in the physical state of the sample [49]. Accordingly, a gel point was identified for the condition in which the variation of *G’* and *G*” exhibited a power-law behavior with a common exponent n (i.e., *G’* and *G”* were proportional to ω ^n^). This criterion implies that *tanδ* (G”/G’) becomes frequency-independent and converges at gel point. Figure 6A shows a multi-frequency plot of *tanδ* as a function of time. The obtained data revealed a general trend for the monotonic decrease of *tanδ* with time, indicative of a progressive suppression of the mobility of the components of the macromolecular network. The crossover point represents a frequency-independent value of *tanδ* (i.e., *tanδ*_(*gel*)_), which is a characteristic parameter for the network formation of a system, in addition to the time at which gelation takes place, i.e., the characteristic gelation time (*t*_(*gel*)_). This suggests that while the gel time (*t*_(*gel*)_) is a natural characteristic parameter of a system, the *tanδ*_(gel)_ value is an additional factor, rooted in its deviation from the theoretical value of 1, which can be attributed to structural features. These two parameters, therefore, will fully define the gelation characteristics of the system at a specified temperature.

To obtain further insights into the macromolecular architecture of the system from the measurements, the crosslink density of the material was estimated from the plateau value of the storage modulus obtained at different times during the TRMS test (data reported in Figure 4), using the following equation:

(4) $$Crosslink\;density=\;\frac{G_{plateau}^{\prime }}{6RT}$$

where *G’_platea_*_u_ is the value of G’ at low frequency, *R* is the gas constant, and *T* is the absolute temperature [50]. The calculated values are reported in Figure 6B and were compared with experimental crosslink densities obtained through the equilibrium swelling of the samples in toluene (red circles), as described in Section 2.3. From these data, it is evident that the crosslink density of the ENR25 network increased with time after gelation and the values of the crosslink density derived from rheological data were in good agreement with those calculated from solvent-swelling measurements. The proposed strategy may foresee further applications in combination with some molecular dynamics approaches, such as the phase-field method used for predicting the evolution of microstructure and properties in materials experiencing thermally activated phase changes [51,52].

## 4. Conclusions

The rheological behavior of ENR containing 25 mol% of epoxy groups (ENR25) was evaluated through TRMS, which made it possible to obtain a clear description of the evolution of the linear viscoelastic response for ENR25 in the melt state (180 °C), associated with changes in the macromolecular architecture that took place during the rheological measurements. Furthermore, the time evolution of the rheological functions revealed a progressive increase of solid-like rheological features of the material, ascribable to thermally activated gelation (free-radical) reactions, which gave rise to molecular motion restrictions, manifest as slower relaxations within the ENR macromolecular network.

The analysis of the derived dynamic master curves demonstrated that conventional single frequency rheological measurements overestimated the modulus.

It was also shown that the procedure of collecting isochronal data at different frequencies from TRMS measurements is a good way to characterize the evolution of network formation through two specific parameters, i.e., the gel time (*t_ge_*_l_) and the *tanδ*(*_gel_*). A good agreement was obtained for the crosslink density after gelation between rheological measurements and solvent-swelling tests.
